# The Long-Term Postural Orthostatic Tachycardia Syndrome Outcomes Survey-Gynecologic Findings: A Cross-Sectional Survey in Young Women

**DOI:** 10.1155/ogi/8872884

**Published:** 2025-07-15

**Authors:** Jeffrey R. Boris, Edward C. Shadiack, Elizabeth M. McCormick, Laura E. MacMullen, Ibrahim George-Sankoh, Frances Fitzgerald, Marni Falk

**Affiliations:** ^1^Jeffrey R. Boris, Moylan, Pennsylvania, USA; ^2^War Related Illness and Injury Study Center, VA New Jersey Healthcare System, East Orange, New Jersey, USA; ^3^Mitochondrial Medicine Frontier Program, Children's Hospital of Philadelphia, Philadelphia, Pennsylvania, USA; ^4^The Ebright Collaborative, Wilmington, Delaware, USA; ^5^Perelman School of Medicine, University of Pennsylvania, Philadelphia, Pennsylvania, USA

**Keywords:** abnormal uterine bleeding, adolescent, autonomic nervous system, dysautonomia, dysmenorrhea, endometriosis, menorrhagia, metrorrhagia, polycystic ovary syndrome

## Abstract

**Objective:** Postural orthostatic tachycardia syndrome (POTS) affects up to 3 million people in the United States. Although 78%–83% of POTS patients are female, gynecologic comorbidity has not been well-studied. We created an online questionnaire to assess outcomes in female patients with POTS formerly followed at a single-center pediatric POTS program.

**Design:** Cross-sectional study.

**Setting:** Single-center pediatric POTS program.

**Population or Sample:** All female patients ≤ 18 years at diagnosis.

**Methods:** We developed and distributed The Long-Term POTS Outcomes Survey with questions about diagnosis, therapy, education, employment, social impact, quality of life (QoL), and gynecologic symptoms and management.

**Main Outcome Measures:** Gynecologic symptoms and QoL.

**Results:** Regular menstrual cycles were seen in 81/167 participants (49.1%). POTS symptoms worsened prior to and during menses in 118/167 subjects (72.4%); hormonal contraceptive therapy helped to control symptoms in 52/110 subjects (50%). Menorrhagia, polycystic ovary syndrome, and endometriosis were not reported in higher numbers compared to the general population.

**Conclusions:** Menstrual flow disorders are not more prevalent in younger females with POTS. Symptoms often worsen perimenstrually, and hormone therapy can help to reduce symptom severity. Further research is needed to better define optimal hormone therapy in suppressing perimenstrual symptoms.

## 1. Introduction

Postural orthostatic tachycardia syndrome (POTS) affects nearly 2.5 million females in the United States, and over three-quarters of POTS patients are female [1]. Although females are disproportionately affected by POTS [1–4], gynecological comorbidities and hormonal influence on symptom presentation and severity are still not well understood. Clinically, female POTS patients have reported worsening symptoms during menstruation [5]. Anecdotally, hormone-based therapy has been used to reduce symptom severity. Despite female predominance, there is limited literature exploring differential presentations based on sex, hormonal influence on disease presentation and management, perimenstrual factors, or gynecologic comorbidities [5–8].

To better understand female comorbidities, perimenstrual influences on POTS symptoms, and other gynecological issues in these patients, we designed a gynecological substudy as part of the Long-Term POTS (LT-POTS) Outcomes Survey in patients with POTS who were diagnosed and followed clinically at a single pediatric center. The LT-POTS survey was created to collect and evaluate multiple aspects of the patient experience from the time of initial diagnosis as well as long-term medical and social concerns.

## 2. Methods

The LT-POTS survey was offered to a group of patients diagnosed with POTS before age 18 years by one of the authors (Jeffrey R. Boris) and/or who were followed in the POTS Program at the Children's Hospital of Philadelphia (CHOP) with a diagnosis of POTS between 2007 and 2018. The diagnosis of POTS was based on a combination of at least 3 months of severe orthostatic intolerance, with significant symptoms spanning cardiovascular, gastrointestinal, and/or neurologic systems, plus sustained increase in heart rate of at least 30 beats per minute from supine to standing during a 10-min standing test without other known etiologies or orthostatic hypotension. The 10-min stand was performed with patients off of all medications that could potentially affect cardiovascular response. Joint hypermobility was assessed using a Beighton scale [9]. Patients and families were contacted by email, and informed consent was obtained electronically, either by the patient or their parent/legally authorized representative, prior to beginning the survey. The survey was designed and administered in the Research Electronic Data Capture (REDCap) [10] system hosted at CHOP.

The LT-POTS survey included questions assessing POTS symptoms, use of prescribed contraceptive therapies and over-the-counter therapies, co-morbid diagnoses, family history, and pregnancy history. Quality of Life (QoL) was evaluated using the Pediatric QoL Inventory (PedsQL) [11] in patients younger than 18 years old at survey administration, and using the 36-Item Short-Form Health Survey (SF- 36) for patients 18 years and older [12]. Scoring for both of these evaluation tools range from 0 to 100, with higher scores correlating to higher health-related QoL. The Menstrual Bleeding Questionnaire (MBQ) [13] was used to evaluate symptoms, including severity, associated with menstruation. Scoring for the MBQ ranges from 0 to 75, with higher scores correlating with higher adverse impact of menstrual bleeding on QoL.

Data were analyzed using median and interquartile range, or using mean and standard deviation when normally distributed. Statistical calculations were performed using Social Science Statistics [14]. The LT-POTS survey and study design was reviewed and approved by the CHOP Institutional Review Board.

## 3. Results

A total of 932 patients were identified to be initially eligible for participation in the LT-POTS study; invitations to participate in the study were sent to 862 patients who had complete and valid contact information. A total of 169 female subjects completed the gynecologic section of the LT-POTS survey and were included for analysis.

The mean subject age was 22.1 years, and the sample was 98% white/Caucasian (Table 1). The PedsQL was completed by 16 patients age < 18 years, while 147 adult subjects completed the SF-36 (Table 2). Pediatric patients had domain scores ranging from 48.0–64.1 on the PedsQL, consistent with moderate impairment across all domains, and an average total score of 52.6. Adult patients also had moderate impairment in most domains of the SF-36, ranging from 57.8–68.4, but had more severe impairment in Role Limitations Due to Physical Health and Energy/Fatigue domains, with scores between 30.6 and 38.8.

All respondents had undergone menarche, with median age of menarche at 12 years (Table 3). A total of 49.1% of patients had regular menstrual cycles, with 34.1% reporting that their cycles were inconsistent. MBQ demonstrated the highest domain score in the Heaviness Score. Pain, QoL, and Irregularity had approximately equal, but lower, scores compared to the Heaviness Score. The median MBQ total score was 13.0 (IQR 8.0–19.3). When restricted to subjects aged ≥ 21 years, the median MBQ total score was 12.0 (data not shown). Up to 72.4% of respondents reported that their POTS symptoms changed during their menstrual cycle, with symptoms seeming worst either premenstrually or during menses, and less severe either early or late postmenstrually (Table 4).

Hormonal contraceptive therapy was used by 66.3% of participants at the time of survey, with a mean duration of use of 6.3 years (Table 5). The majority of those using hormonal contraceptive therapy (73.6%) used either a combined oral contraceptive or a progestin-only intrauterine device (IUD). Multiple reasons were given for use, with 81.8% of subjects trying to regulate or stop their menses. Nearly half of patients reported dysmenorrhea as a cause for hormonal contraceptives, while 38.2% specifically used them to manage POTS symptoms. In patients using hormonal contraceptives to manage POTS symptoms, 50% felt that there was at least some symptomatic improvement, while another 40.4% felt that there was no change.

Various alternative therapies were reportedly used to reduce menstrual symptoms (Table 6). Licorice root and ginger, the most commonly used therapies, were effective at reducing menstrual symptoms 56% and 44% of the time, respectively.

**Figure 1:**
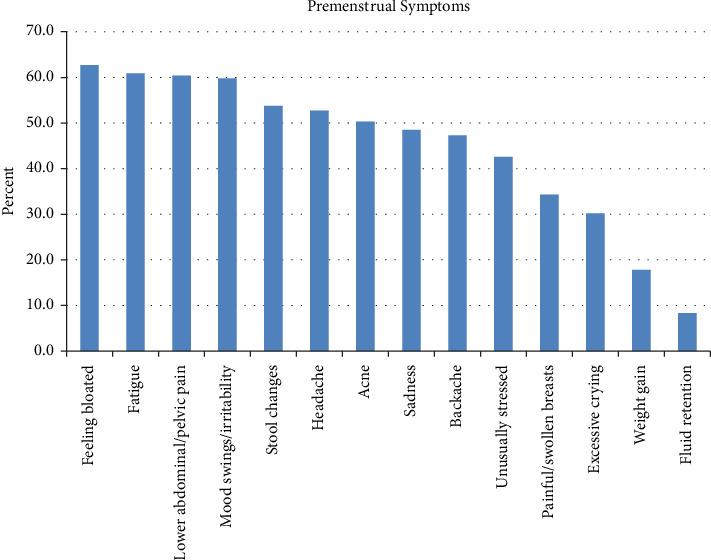
Premenstrual symptoms.

Multiple premenstrual symptoms were listed (Figure 1). At least 50% of patients noted bloating, fatigue, abdominal/pelvic pain, mood swings/irritability, stool changes, headache, and acne. The stool changes included either more frequent (34.1%) or softer stools (33.0%), while the rest had less frequent (15.4%), more painful (13.2%), or harder stools (4.4%).

**Figure 2:**
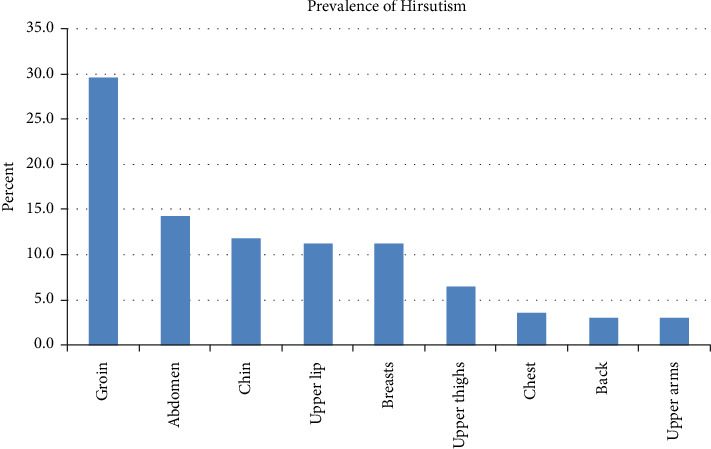
Prevalence of Hirsutism.

The tendency to grow coarse dark hair was reported in 29.6% of patients, including at the groin, and in at least 10% of patients at other areas, including the abdomen, chin, upper lip, and breasts (Figure 2).

**Figure 3:**
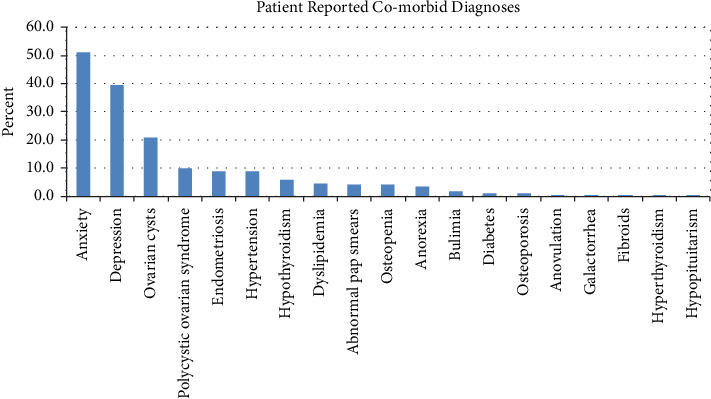
Patient reported co-morbid diagnoses.

**Figure 4:**
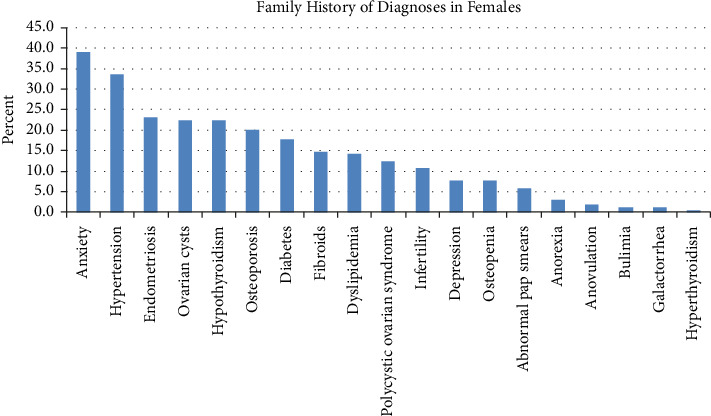
Family history of diagnoses in females.

Comorbid anxiety and depression diagnoses were reported in 50.9% and 39.6% of subjects, respectively (Figure 3). Ovarian cysts were reported in 20.7% of respondents, 10.1% had a diagnosis of polycystic ovary syndrome (PCOS), and 8.9% reported endometriosis at the time of survey. Those same three diagnoses were also seen in female family members, with 23.1% having endometriosis, 22.5% having ovarian cysts, and 12.4% having PCOS, plus another 14.8% having uterine fibroids (Figure 4). Anxiety and depression were seen in 39.1% and 7.7% of family members, respectively. Multiple other diagnoses were reported in the subjects at rates below 10%, and in their family members in rates below 25%.

## 4. Discussion

### 4.1. Main Findings

Our study shows that females with POTS have moderate or severe impairment of QoL. Although symptoms of abnormal uterine bleeding (AUB) do not appear to be seen in higher frequency in POTS patients in our study than the general public, POTS symptoms appear to worsen perimenstrually. It is notable that although secondary amenorrhea is more frequent than controls in an older cohort of women with POTS (mean age 33 years), AUB was not found to be significantly different from controls [5]. However, that same study demonstrated that the prevalence of gynecologic abnormalities was higher in these patients. As well, up to 60% of patients experienced symptoms of premenstrual syndrome.

### 4.2. Strengths and Limitations

This study's strengths include its size and symptom duration, plus the comprehensive review of the scope of gynecologic and co-morbid disorders. As a cross-sectional survey, though, it is prone to the biases of this study design, including recall bias and placebo effect. The average time from symptom onset to survey completion was nearly 10 years, so specific clinical aspects may not be adequately recalled. As well, it is possible that patients with worse or persistent symptoms may have been more likely to complete the survey versus those who were clinically better. Another limitation is the younger age relative to risk of increased bleeding concerns. Additionally, our POTS patients were diagnosed with a 30 beat-per-minute threshold by 10-min standing test, differing from published guidelines using 40 beats-per-minute by tilt table test in patients aged 12–19 years [15]. However, patients had been diagnosed in our clinic using the lower threshold since 2007, prior to publication of those criteria. Furthermore, tilt testing induces a higher overall heart rate versus standing test [16], and symptomatology is not significantly different using either threshold [17].

### 4.3. Interpretation

Female predominance of POTS patients is well-documented [1–4]. Other disorders disproportionately affecting women include autoimmune diseases [18] (rheumatoid arthritis, Sjögren's disease, and systemic lupus erythematosus, and multiple sclerosis [19, 20]), migraine [20], and psychiatric disorders [21]. Myalgic encephalomyelitis/chronic fatigue syndrome, with symptoms similar to POTS [22, 23], has female predominance [24]. Further complicating this is the tendency that females are less likely to be taken seriously when reporting medical concerns [2, 25].

Our patients were relatively young, most were unmarried (Table 1), and there were few pregnancies (Table 7). However, they provided important information regarding gynecologic findings. About one-half of patients had regular menstrual cycles (Table 3), and another third had inconsistent cycles, reporting cycle durations between < 22 and > 31 days. Typically, normal cycle length ranges from 22 to 36 days in 95% of women, and 42.5% have cycle variability of > 7 days [26], with our findings similar to that of the general population. In the MBQ validation study, 60.3% of patients reported irregular cycles, with 53% reporting heavy menstrual bleeding [13]. In our study, the median MBQ total score of 13.0 is similar to the original validation study group with normal menses (10.6), while significantly different from the group with heavy menstrual bleeding (30.8). In the original MBQ study, irregular bleeding correlated with a mean MBQ total score of 12.7, also similar to subjects without uterine bleeding problems. Notably, although IUDs are associated with irregular menstrual cycles, there was no difference in reported irregular menses between those patients using IUDs versus those who did not (*p* value 0.07, data not shown). Thus, although some POTS patients report heavy bleeding, overall, they are more likely to have normal menstrual flow. The adolescent version of the MBQ demonstrated scores of 19.6 for groups with normal menses and 29.8 for those with heavy bleeding [27], also suggesting that POTS patients are similar to the general population.

Most respondents reported worsening POTS symptoms during premenstrual and menstrual phases. Similar findings were previously reported, demonstrating that lightheadedness in adults with POTS was worst during menses [5].

However, our findings were contrary to those of a 2015 study, in which supine and standing data on POTS patients were analyzed relative to menstrual phase [7]. They found greater presyncope in patients in early follicular (early postmenstrual) phase versus mid-luteal (premenstrual) phase. However, blood pressure was elevated in mid-luteal phase versus early follicular phase.

Coupal, et al., suggested that estrogen, progesterone, and other non-sex hormones, lead to hypovolemia, decreased vasoconstriction, and poor cerebral autoregulation in POTS patients [8]. With two-thirds of our subjects using hormonal contraceptives, and over one-third specifically using them to modulate POTS symptoms, there is recognition that perimenstrual effects may be mitigated by hormonal intervention. Estrogen levels spike during late follicular phase, both estrogen and progesterone increase during mid-luteal phase (with relatively higher progesterone levels), and both decrease in late luteal phase (premenstrual), suggesting that progesterone, or relatively lower estrogen, may help to reduce POTS symptoms in early and late postmenstrual phases. At least half of our patients reported symptom reduction with contraceptive therapy. Our study did not evaluate relative effectiveness of these formulations, requiring future research to evaluate differences in effectiveness.

Hirsutism was seen in around 10% of patients (Figure 2), similar to its prevalence in the general population [28]. Hirsutism is often associated with PCOS. In our series, PCOS occurred in 10% of patients (Figure 3), and occurs in 4%–20% of women worldwide [29]. This is in line with that of the general population, as is our finding of over 12% of participants reporting family members with PCOS (Figure 4). Similarly, nearly 9% of patients having endometriosis (Figure 3) are also consistent with its reported prevalence of 6%–10% [30]. Conversely, a prior survey found that endometriosis occurred more frequently in women with POTS (20%) versus controls (5%); the mean age of their subjects was 33 years, much older than in our study [5]. They also found increased AUB (14% vs. 4% in controls), plus the presence of uterine fibroids, galactorrhea, and ovarian cysts, in women with POTS versus controls, likely due to the older age of the evaluated sample. As well, although our study included patients whose POTS symptoms started at or below age 18 years, our findings may not translate to those patients whose symptoms also started at an older age.

In our younger population with few reported pregnancies (Table 7), we cannot draw any conclusions about pregnancy in the setting of POTS. Since 2005, research on pregnancy and POTS has included observational case reports and series [31]. Significant POTS symptom variability during pregnancy has been seen, with some patients reporting improvement and others reporting worsening symptoms [32]. Hyperemesis gravidarum was described in 59% of patients, although that study was also survey-based and thus unclear whether “hyperemesis gravidarum or severe vomiting” was defined for participants [33]. However, similar to our study, 40% of patients had worsening of POTS symptoms during pregnancy.

In assessing QoL, both younger and older patients demonstrated moderate impact of disease on their daily life and health, although physical health, energy and fatigue, and total scores in the SF-36 tended to be more severe in older patients as compared to those of younger patients in the PedsQL (Table 2). These questions were asked in the context of their overall POTS symptoms, and not specifically linked to the gynecological portion of the survey. Notably, while the adolescent version of the MBQ has had good validity when compared to the PedsQL [27], the MBQ has only low to moderate correlation with the SF-36 [14], and may not be predictive of effect on QoL.

Although it is unclear why females are more commonly diagnosed with POTS, hormonal mechanisms have been postulated. In general, females demonstrate increased vasodilation compared to males [34], and research has shown that sex hormones have receptors in the vasculature directly affecting vascular tone [35, 36] and the brain [37] modulating autonomic response. This may explain the higher female predominance, as well as why hormonal contraceptive therapy helps reduce POTS symptoms.

Further studies, including use of controlled trials, are needed to evaluate effectiveness of specific subtypes of hormonal therapy, such as low estrogen-containing treatments. This will help better guide providers and patients to further control perimenstrual symptoms of POTS and may provide insight into reasons for the widely disparate sex distribution of POTS patients.

## 5. Conclusions

We show that females with POTS did not demonstrate increased AUB but have significant menstrual irregularity. PCOS and endometriosis do not occur more frequently in these patients. POTS symptoms are worse during premenstrual and menstrual phases, with hormonal contraception therapy reducing symptom variability and severity in some patients.

## Figures and Tables

**Table 1:** Demographics (*N* = 169).

	Mean (SD)
Age	22.1 (3.4)

**Race/ethnicity**	***N* (%)**

White	166 (98.2)
Black/African-American	1 (0.6)
Asian	1 (0.6)
Native American	1 (0.6)
Hispanic	5 (3.0)

	**Mean (SD)**

Years from onset of POTS to present	9.7 (3.4)

**Table 2:** Quality of life.

	Scale 0–100^∗^, *N* = 16	Mean (SD)
PedsQL (age < 18)	Physical functioning	48.0 (22.8)
	Emotional functioning	50.9 (18.6)
	Social functioning	64.1 (20.5)
	School functioning	50.0 (22.0)
	Total score	52.6 (16.9)

	**Scale 0–100** ^**∗**^ **, *N* = 147**	**Mean (SD)**

SF-36 (age 18+)	Physical functioning	68.4 (25.0)
	Role limitations due to physical health	38.8 (40.2)
	Role limitations due to emotional problems	57.8 (39.8)
	Energy/fatigue	30.6 (19.7)
	Emotional well-being	61.3 (19.2)
	Social functioning	61.0 (26.4)
	Pain	58.7 (23.8)
	Total score	38.6 (19.8)

^∗^Higher scores on the PedsQL and SF-36 are associated with better health-related QoL.

**Table 3:** Menarche and the menstrual bleeding questionnaire (MBQ).

	*N* (%)
Have you begun having your menstrual period?	167 (100)

	**Median (IQR)**

How old were you when you had your first menstrual period (years)?	12 (11–13)

	***N* (%)**

Regular menstrual cycles	81 (49.1)

**How long is your menstrual cycle?**	***N* (%)**

< 22 days	15 (9.1)
22–26 days	19 (11.6)
27–31 days	58 (35.4)
> 31 days	16 (9.8)
Inconsistent	56 (34.1)

**MBQ domain scores**	**Median (IQR)**

MBQ heaviness score (0–36)	5 (3–7.3)
MBQ pain score (0–3)	2 (1.0–2.0)
MBQ QoL score (0–29)	2 (0–6.0)
MBQ irregularity score (0–7)	3 (1.0–4.0)
MBQ total score (0–75)	13.0 (8.0–19.3)

**Table 4:** Perceived effect of the menstrual cycle on POTS *N* (%).

	Yes	No
Changes in POTS symptoms throughout the course of the menstrual cycle?	118 (72.4)	45 (27.6)

	**Time during cycle that POTS symptoms are WORST:**	**Time during cycle that POTS symptoms are LEAST:**

Premenstrual (1–10 days before flow)	28 (24.3)	8 (7.1)
Menstrual (day 1–5)	85 (73.9)	4 (3.6)
Early postmenstrual (day 6–14)	2 (1.7)	29 (25.9)
Late postmenstrual (day 15–22)	0	71 (63.4)

**Table 5:** Utilization of contraceptive therapy *N* (%), except where noted.

Currently using hormonal contraceptive therapy	110 (66.3)
Years of using hormonal contraceptive therapy; mean (SD)	6.3 (3.3)
Type of contraceptive (110 replies)	
Combined oral contraceptive	59 (53.6)
Depot medroxyprogesterone	9 (8.2)
Progestin-only mini pill	9 (8.2)
Contraceptive patch	1 (0.9)
Vaginal ring	3 (2.7)
Progestin-only intrauterine device	22 (20.0)
Implantable rods	6 (5.5)
Reason for starting hormonal contraceptive therapy (110 replies)	
Regulate menses	57 (51.8)
Stop menses	33 (30.0)
Dysmenorrhea	52 (47.3)
Birth control	34 (30.9)
Controlling POTS symptoms	42 (38.2)
Contraceptive effectiveness for prescribed purpose	97 (89.0)
Contraceptive effect on POTS symptoms	
Much better	25 (24.0)
Somewhat better	27 (26.0)
No changes	42 (40.4)
Somewhat worse	4 (3.8)
Much worse	6 (5.8)

**Table 6:** Use of alternative therapies.

Therapy	Ever used; *N* (% of total)	Effective at alleviating symptoms; *N* (% of ever used)
Black cohosh	1 (0.6)	1 (100)
Saw palmetto	2 (1.2)	2 (100)
Peony	1 (0.6)	1 (100)
Licorice root	25 (14.8)	14 (56)
Flaxseed	14 (8.3)	10 (71)
DHEA	4 (2.4)	4 (100)
Evening primrose oil	4 (2.4)	2 (50)
Soy	1 (0.6)	1 (100)
St. John's Wort	0	0
Gingko Biloba	1 (0.6)	1 (100)
Ginger	25 (14.8)	11 (44)
Chasteberry	5 (3.0)	2 (40)

**Table 7:** Pregnancy and POTS *N* (%).

Survey question	Yes
Ever pregnant?	6 (3.6)
How many times pregnant?	
1	2 (33.3)
2	4 (66.7)

	Yes
Were there complications with the pregnancies?	5 (83.3)
Pregnancy complications	
Hyperemesis	3 (60)
Miscarriage	2 (40)
Worse POTS symptoms	2 (40)

## Data Availability

The data that support the findings of this study are available from the corresponding author upon reasonable request.
